# Efficacy and safety of micafungin for the treatment of patients with proven or probable invasive aspergillosis: A non-comparative, multicenter, phase IV, open-label study

**DOI:** 10.1097/MD.0000000000009443

**Published:** 2017-12-29

**Authors:** Yu Ji, Yongping Song, Fang Zhou, Ting Liu, Ming Jiang, Xielan Zhao, Xiaojun Huang

**Affiliations:** a Beijing United Family Hospital; b Peking University People's Hospital, Beijing; c HeNan Cancer Hospital, Zhangzhou; d The Affiliated Cancer Hospital of Zhengzhou University, Zhengzhou; e General Hospital of Jinan Military Area, Jinan; f West China Hospital of Sichuan University, Chengdu; g The First Affiliated Hospital of Xinjiang Medical University, Urumqi; h Xiangya Hospital, Central South University, Changsha, China.

**Keywords:** efficacy, invasive aspergillosis, invasive fungal disease, micafungin, overall treatment success, safety

## Abstract

**Introduction:**

Few studies have assessed the efficacy and safety of micafungin in patients with proven or probable invasive aspergillosis (IA). This was the aim of the current study, which was conducted in 22 hospitals in China, where micafungin was approved for treatment of IA in 2006.

**Methods:**

This was a non-comparative, phase IV open-label study (NCT02646774). Eligible patient were adults with proven or probable IA. Efficacy endpoints included rates of overall treatment success (primary endpoint) and clinical improvement, fungal clearance, mortality, and the site of *Aspergillus* infection (all secondary endpoints). Safety endpoints included incidences of treatment-emergent adverse events (TEAEs), serious AEs (SAEs), and adverse drug reactions (ADRs). These endpoints were reported descriptively with associated 95% confidence intervals (CI); no hypotheses were tested.

**Results:**

The study was discontinued early due to low patient recruitment, which did not allow for the planned sample size to be reached. In total, 68 patients were enrolled: 42 into the full analysis set (for efficacy) and 61 into the safety analysis set. All patients were Han Chinese; the majority were male (n = 26; 61.9%) and ≤60 years of age (n = 35; 83.3%). Rates of overall treatment success, clinical improvement, fungal clearance, and mortality were 45.2% (n = 19/42; 95% CI: 29.85–61.33); 59.5% (n = 25/42; 95% CI: 43.28–74.37), 80.0% (n = 4/5; 95% CI: 28.36–99.49), and 7.1% (n = 3/42; 95% CI: 1.50–19.48), respectively. All patients were diagnosed with pulmonary *Aspergillus* infection. Overall, 155 TEAEs and 8 SAEs were reported by 37 (60.7%) and 7 (11.5%) patients. The most common TEAEs were decreased platelet count and fatigue (both n = 5; 8.2%) and the most common SAEs were intracranial hemorrhage and lung infection (n = 3; 4.9% and n = 2; 3.3%). Eight ADRs (n = 6; 9.8%) were reported but all were completely remitted or remitting during follow-up.

**Conclusions:**

Results suggest that micafungin is efficacious and well-tolerated in patients with proven or probable IA in China. However, these findings should be interpreted with care, due to the small number of patients included in this study. Further comparative trials should be used to confirm the efficacy and safety of micafungin in patients with proven or probable IA.

## 1 Introduction

Invasive fungal disease (IFD) caused by *Aspergillus* species (invasive aspergillosis; IA) is a significant cause of morbidity and mortality,^[1–3]^ particularly in immunocompromised patients undergoing chemotherapy or transplantation.^[1,2,4,5]^ The incidence of IA has increased substantially in recent years, in part associated with the introduction of fluconazole prophylaxis to prevent *Candida* infections.^[6,7]^ The most common *Aspergillus* spp. isolated from cases of IA are *Aspergillus fumigatus*, *Aspergillus flavus*, *Aspergillus niger*, and *Aspergillus terreus*, although up to 7% remain unidentified.^[8,9]^ The majority of IFDs due to *Aspergillus* spp. are limited to the lungs, respiratory tract, and sinuses.^[9,10]^ Although other organs (e.g., heart, kidneys, liver, and pancreas) can also be affected.^[9]^

IA can be defined as proven, probable, and possible.^[11]^ Proven cases should be based on histopathology, cytopathology, or direct microscopic examination, and positive *Aspergillus* culture test of specimens from normally aseptic sites. Probable cases are those that meet criteria within 3 categories: host factors, clinical manifestations (symptoms, signs, and radiological features), and mycological evidence.^[3,11]^ The overall incidence of proven IA in patients in intensive care units is thought to be up to 17%, with an associated mortality rate of up to 79%.^[12]^ However, the incidence rate of IA may vary depending on local epidemiology and host risk factors, as well as the quality of air control in hospital settings.^[13]^

Micafungin is an echinocandin with a broad-spectrum of activity against *Aspergillus* spp.^[14]^ The efficacy and safety of micafungin when used as prophylaxis or empirical therapy for IFDs has been shown in randomized, multicenter trials (including 1 conducted in China).^[15–17]^ In these studies, the overall treatment success rates (i.e., the absence of suspected, proven, or probable invasive fungal infection) at the end of micafungin treatment were similar to those of active comparators (fluconazole or itraconazole); similar tolerability was observed with micafungin and fluconazole,^[15]^ but improved overall tolerability was observed for micafungin compared with itraconazole.^[16,17]^ In each study, fewer cases of probable or proven breakthrough IA (proven or probable disease with onset of symptoms on day 3 or later after initiation of antifungal therapy^[3]^) were reported in patients treated with micafungin, compared with the comparator treatments.^[15–17]^

The majority of studies of micafungin in patients with IA are limited to case reports, as discussed by Enoch et al.^[18]^ Indeed, few studies have evaluated the efficacy and safety of micafungin in patients with proven or probable IA.^[19–21]^ Micafungin demonstrated similar efficacy compared with caspofungin (overall response rates: 42.4% vs 46.7%, respectively) in a randomized, double-blind, multicenter trial of 120 patients with proven or probable IA conducted in Japan, and similar overall tolerability was also observed (adverse events [AEs] reported by 38.3% vs 41.7% of patients).^[21]^ In the other trials, both of which were non-comparative, patient numbers were low (n ≤ 29 for all efficacy assessments), and 1 trial was discontinued early due to issues with enrollment.^[19,20]^ However, across both of these trials, overall response rates of up to 50.0% were observed in patients who received micafungin as monotherapy.^[19,20]^

Micafungin was approved for the treatment of infectious diseases caused by *Aspergillus* spp. in China in 2006 (it is approved for the treatment of invasive candidiasis but not IA in Europe and the United States).^[22,23]^ Post-marketing data reported in Chinese patients with IA have shown favorable treatment response rates for micafungin compared with fluconazole (66.7% vs 44.4%, respectively), and an acceptable tolerability profile.^[24]^

The objective of the current study was to evaluate the efficacy and safety of micafungin for the treatment of patients with proven or probable IA.

## 2 Methods

### 2.1 Study design and treatment

This was a non-comparative, multicenter, phase IV, open-label study (Astellas protocol number: ACN-MA-MYC-IA-2012; clinicaltrials.gov identifier: NCT02646774), conducted in 22 hospitals in China. The date of first enrollment was March 1, 2014 and the last evaluation was completed on June 26, 2015. All eligible patients were treated once-daily (OD) with micafungin via intravenous infusion; the dosage used was at the discretion of the treating physician, ranging from 50 to 300 mg/d. Patients were treated for up to 12 weeks according to disease severity; the treatment duration was calculated on the day patients were enrolled and first received micafungin (Day 0).

### 2.2 Patients

Eligible patients were adults ≥18 years of age, with proven or probable infections caused by *Aspergillus*, according to European Organization for Research and Treatment of Cancer/Invasive Fungal Infections Cooperative Group/National Institute of Allergy and Infectious Diseases Mycoses Study Group criteria.^[3]^ Patients highly suspected to have IA were also enrolled and received micafungin on Day 0; however, these patients only continued in the study if they were diagnosed with proven or probable IA on the third day after their first dose of micafungin.

Patients were excluded due to: lack of a negative pregnancy test prior to the study; unwillingness to use reliable methods of contraception throughout the study; receipt of any echinocandin or enrollment in another clinical study within 1 month prior to enrollment into the current study; aspartate aminotransferase/alanine aminotransferase levels >5 times the upper limit of normal (ULN); total bilirubin level >2.5 times the ULN; blood urea nitrogen/creatinine level >3 times the ULN; being human immunodeficiency virus-positive; history of hypersensitivity or any serious reaction to any echinocandin; life expectancy of <1 month; or previous enrollment in the current study. Patients were also excluded if the investigator considered them unlikely to comply with the protocol-scheduled visits, or if they had a history of non-compliance in other trials.

Written informed consent was obtained from all patients. The study was approved by local ethics committees, who were informed of all serious AEs (SAEs) which occurred during the study. The study was conducted in accordance with the principles outlined in the Declaration of Helsinki, Good Clinical Practice, and all applicable laws and regulations.

### 2.3 Endpoints and assessments

#### 2.3.1 Efficacy (assessed each week after initiation and at completion of micafungin treatment)

The primary endpoint was the overall treatment success rate (derived by calculating the proportion of patients with a complete or partial response; full definitions for these response criteria are shown in Table 1).^[25]^

**Table 1:**
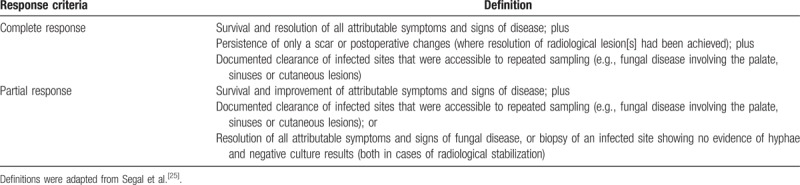
Full definitions of complete and partial response.

Secondary endpoints included clinical improvement rate (the proportion of patients with improvement or complete resolution of symptoms and signs), fungal clearance rate, mortality rate, and the site of *Aspergillus* infection. The fungal clearance rate represented the proportion of patients with confirmed clearance (by negative result for fungal microscopy or culture) or assumed clearance (by complete removal of clinical symptoms and signs, if no repeated sampling was accessible).

#### 2.3.2 Safety

Safety endpoints (assessed at all visits) included the incidence and severity of treatment-emergent AEs (TEAEs; reported according to the Medical Dictionary for Regulatory Activities, version 16.0) and SAEs, including adverse drug reactions (ADRs), considered by the investigator to be related to micafungin treatment. AEs of special interest included those associated with hepatobiliary and renal function. Vital signs, clinical laboratory results, and exposure to micafungin (time and dose) across the treatment period were also assessed.

#### 2.3.3 Follow-up

Follow-up, comprising physical examination, laboratory testing, and other assessments, was performed up to 2 weeks after completion of micafungin treatment.

### 2.4 Sample size

The planned sample size was 120, based on the following formula: *n* = (*μ*^2^_*α*/2_*π*[1 − *π*])/*δ*^2^, and considering a 20% dropout rate, where *π* is the overall success rate, assumed to be 50%, based on previously reported data^[26]^; *δ* is the acceptable 95% CI precision, defined as 10%; and *μ*_*α*/2_ is the 1 – *α*/2 percentile of the standard normal distribution, which is 1.96 when *α* = 0.05.

### 2.5 Analysis subsets

Efficacy was evaluated in the full analysis set (FAS; primary population) and the per protocol set (PPS; secondary population). All enrolled patients who received ≥1 dose of micafungin and had a post-baseline efficacy assessment were included in the FAS; all patients who had been enrolled and received a complete course of micafungin for ≥2 weeks (or ≥4 weeks in patients with hematological disease) were included in the PPS. Patients who received ≥1 dose of micafungin and had a post-baseline safety assessment were included in the safety analysis set (SAS).

### 2.6 Statistical analyses

All pre-specified endpoints were reported descriptively, with associated 95% confidence intervals (CI) calculated for efficacy assessments. No hypotheses were tested.

Post-hoc subgroup analyses (multivariate logistic regression analyses including covariates as independent variables, unless stated) were conducted to assess the impact of the following stratification factors on the overall treatment success rate (FAS, PPS): age (<60 and ≥60 years), sex (male and female), initial dose (continuous variables; 100, 150, 200, and 300 mg), and granulocyte count at enrollment (<0.5 × 10^9^/L and ≥0.5 × 10^9^/L). Similar analyses were performed to assess the impact of the following stratification factors on the incidence of ADRs using data from the SAS: age (<60 and ≥60 years), sex (male and female), and total dose (<1000 mg and ≥1000 mg). Odds ratios (ORs) were calculated and a significance level of *P* < .05 was required to conclude that there was a significant difference. The maximum likelihood estimates for logistic regression were based on iterative methods; therefore, no close form formulae were used.

All analyses were conducted using Statistical Analysis Software version 9.2 (JMP, Marlow, Buckinghamshire, United Kingdom).

## 3 Results

### 3.1 Patient demographics and characteristics

This study was terminated early due to fewer patients being recruited than expected, which did not allow for the planned sample size of 120 to be reached.

**Figure 1:**
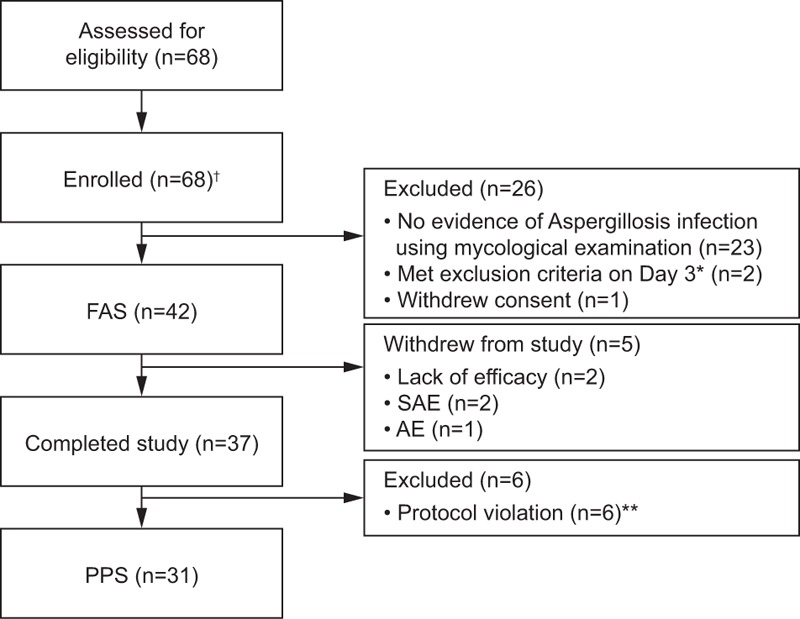
Patient disposition throughout the study. ^†^61 patients received treatment with ≥1 dose of micafungin and had a post-baseline safety assessment, these patients were included in the SAS. ∗On day 3, 1 patient's serum total bilirubin exceeded 2.5 time the upper limit of normal, the other had a life expectancy of <1 month. ∗∗Four of these patients also had elevated liver function. AE = adverse event, FAS = full analysis set, IA = invasive aspergillosis, PPS = per protocol set, SAE = serious AE, SAS = safety analysis set.

Overall, 68 patients were enrolled in the study and 61 patients received treatment with ≥1 dose of micafungin (Fig. 1); these patients were included in the SAS. Forty-two eligible patients with proven or probable IA were included in the FAS, of which 5 discontinued treatment. Of the 37 patients who completed the study, 6 were excluded for protocol violation, resulting in 31 patients being included in the PPS. Of the 26 patients enrolled who were excluded from the efficacy analyses, the majority (n = 23) were excluded because of no established proven or probable *Aspergillus* infection 3 days after their first dose of micafungin.

Patient demographics and characteristics in the FAS are shown in Table 2. All patients were Han Chinese; the majority were male (n = 26; 61.9%) and ≤60 years of age (n = 35; 83.3%). All patients included in the FAS had ≥1 positive mycological examination at baseline. Medical history included treatment with antifungal or immunosuppressive drugs, and neutropenia 2 weeks prior to baseline; and abnormal chest computed tomography (CT) scan, malignant blood disease, and invasive mycotic infection at baseline.

**Table 2:**
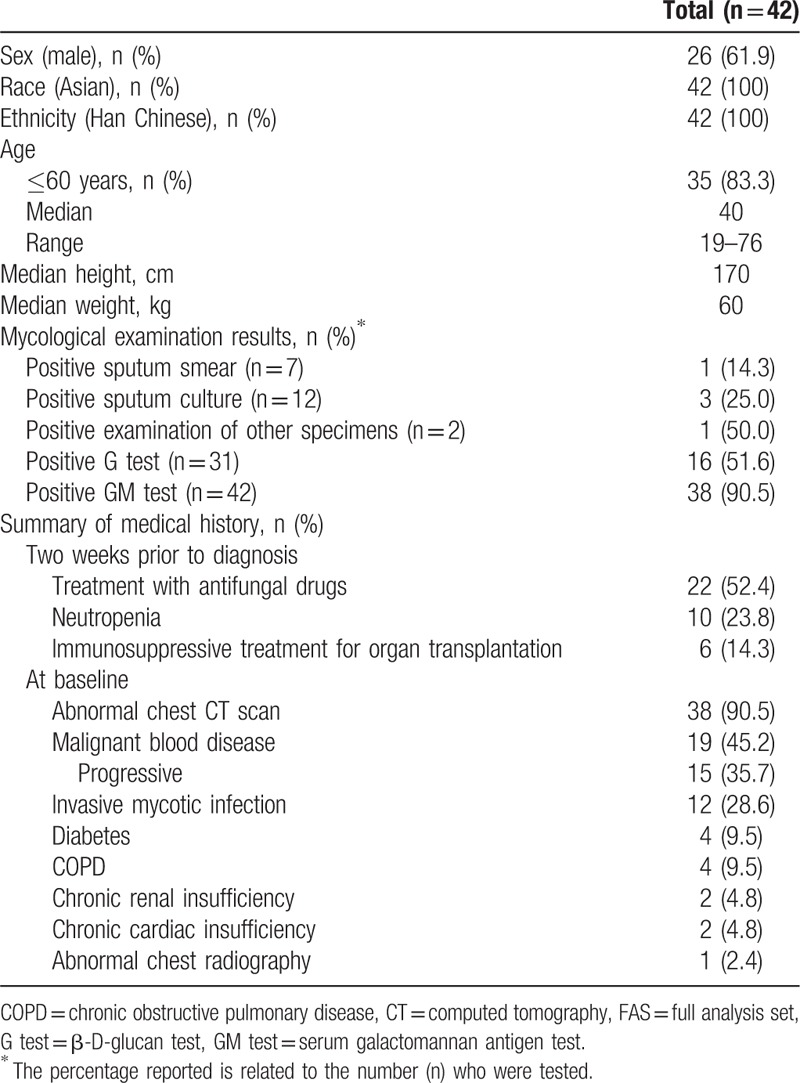
Patient demographics and characteristics (FAS).

### 3.2 Efficacy

#### 3.2.1 Primary endpoint

The overall treatment success rate was 45.2% (19/42 patients; 95% CI: 29.85–61.33) in the FAS and 54.8% (17/31 patients; 95% CI: 36.03–72.68) in the PPS (Table 3). All patients considered to have treatment success in the FAS and the PPS had a partial response to treatment, rather than a complete response.

**Table 3:**
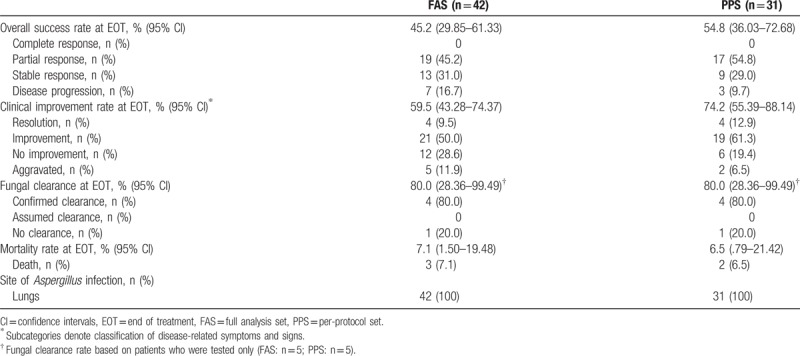
Summary of efficacy assessments.

#### 3.2.2 Secondary endpoints

The clinical improvement rate was 59.5% (25/42 patients; 95% CI: 43.28–74.37 in the FAS and 74.2% (23/31 patients; 95% CI: 55.39–88.14) in the PPS (Table 3). The majority of these patients (21 [50%] in the FAS and 19 [61.3%] in the PPS) experienced an improvement in symptoms and signs. The fungal clearance rate at end of treatment was 80.0% (4/5 patients) in the FAS and the PPS (95% CI: 28.36–99.49) (Table 3). Each case of fungal clearance reported was confirmed clearance, as opposed to assumed clearance. The mortality rate was 7.1% (3/42 patients; 95% CI: 1.50–19.48) in the FAS and 6.5% (2/31 patients; 95% CI: 0.79–21.42) in the PPS (Table 3). All patients in the FAS and PPS were diagnosed with pulmonary *Aspergillus* infection (Table 3).

### 3.3 Safety

Overall, 155 TEAEs and 8 SAEs were reported by 37 (60.7%) and 7 (11.5%) patients, respectively during the treatment period (Tables 4 and 5). The most common TEAEs were decreased platelet count and fatigue, both occurring in 5 (8.2%) patients; the most common SAEs were intracranial hemorrhage and lung infection, occurring in 3 (4.9%) and 2 (3.3%) patients, respectively. Overall, there were 6 deaths resulting from SAEs during the study.

**Table 4:**
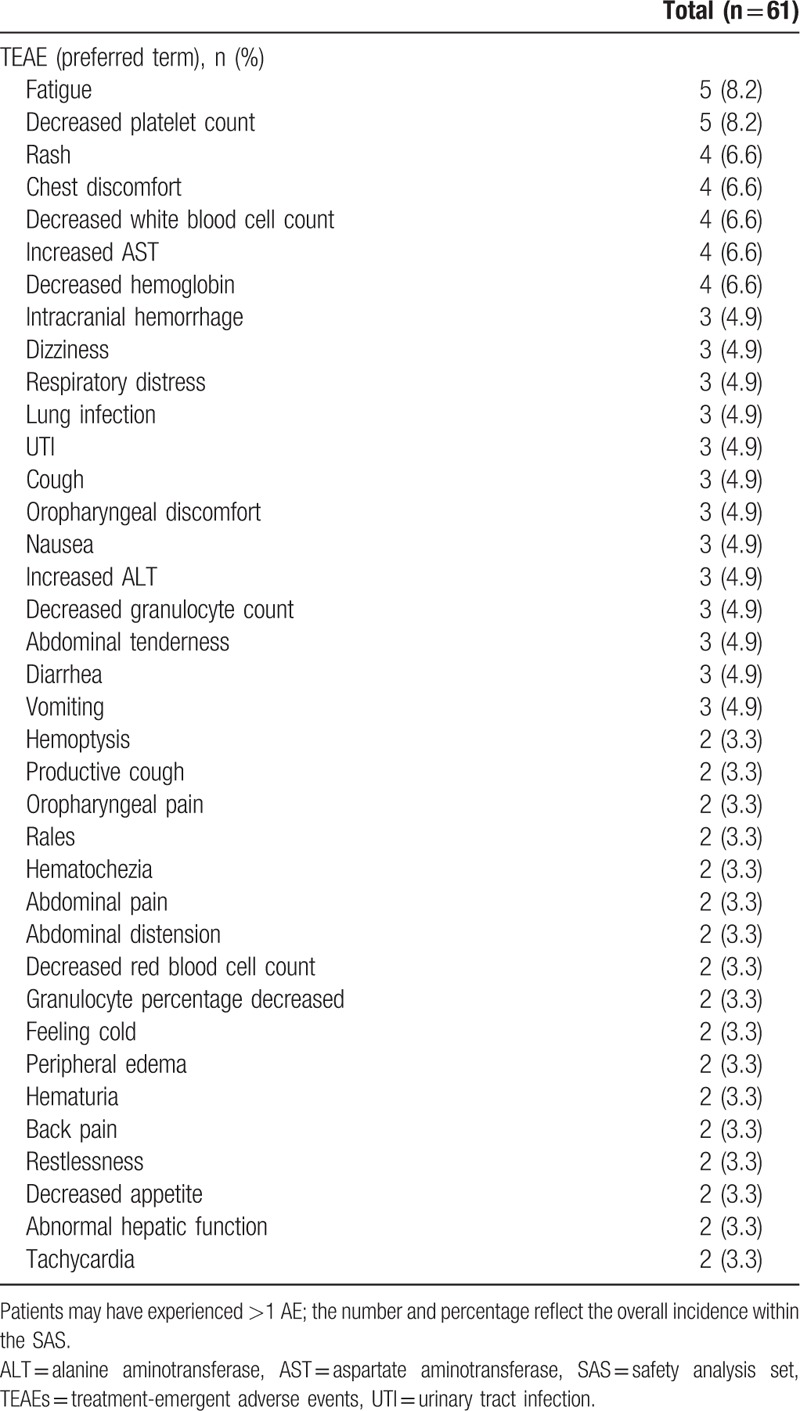
TEAEs occurring in ≥2% of patients in the SAS.

**Table 5:**
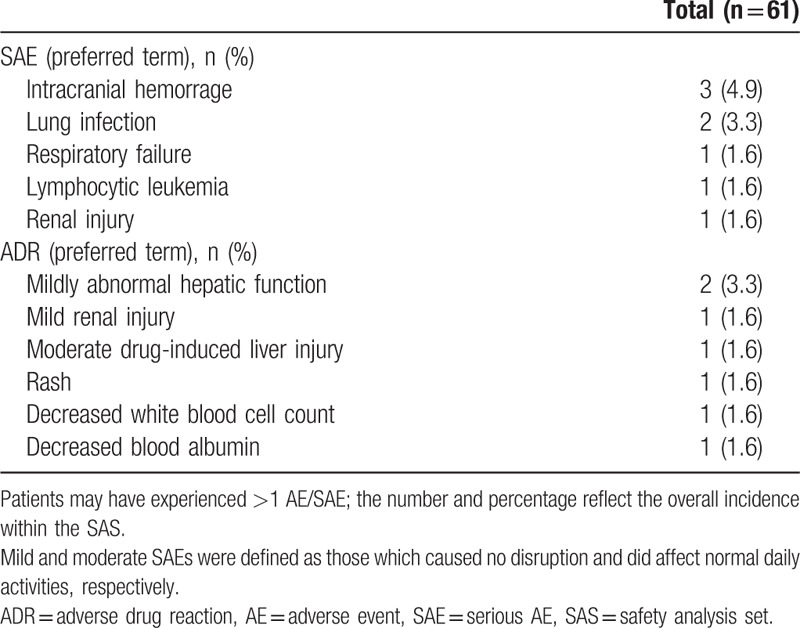
Summary of SAEs; and ADRs considered related to micafungin treatment (both SAS).

In total, 7 ADRs were observed in 6 (9.8%) patients (Table 5). The most common was mildly abnormal liver function (n = 2; 3.3%); mild renal injury, moderate drug-induced liver injury, rash, decreased white blood cell count, and decreased blood albumin were observed in 1 patient each (1.6%). Hepatobiliary and renal AEs were experienced by 3 (4.9%) and 5 (8.2%) patients, respectively. All ADRs (considered related to micafungin treatment) were completely remitted or remitting during the follow-up period.

Vital signs and clinical laboratory results (Table 6) were generally within normal ranges at the end of treatment, although 16 (34.8%), 19 (37.3%), and 20 (37.0%) patients, respectively, had abnormal respiratory rate, aspartate aminotransferase (AST), and urea nitrogen levels at the end of treatment, after reporting normal rates/levels before treatment. The mean exposure to micafungin was 18.44 days (standard deviation [SD]: ±21.50); the mean daily and total doses were 168.22 mg (SD: ±47.96) and 3372.46 mg (SD: ±3991.25], respectively (Table 6).

**Table 6:**
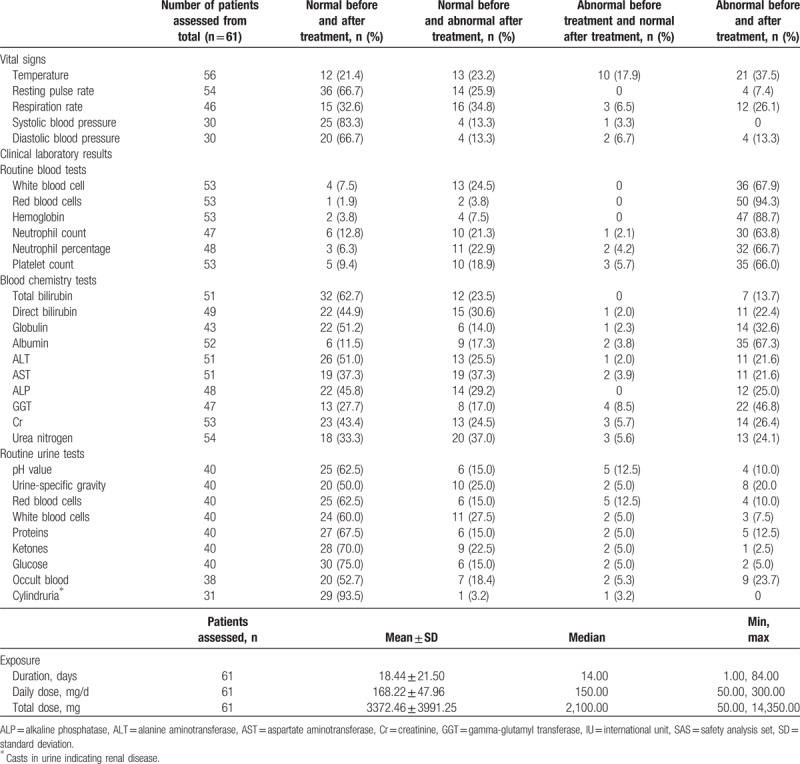
Vital signs, clinical laboratory results, and time of exposure to micafungin across the treatment period (SAS).

### 3.4 Subgroup analyses

According to the data from the multivariate logistic regression models, the initial dose of micafungin was the only stratification factor that had a statistically significant impact on the overall treatment success rate in the FAS (Table 7); no statistically significant differences were reported in the PPS.

**Table 7:**
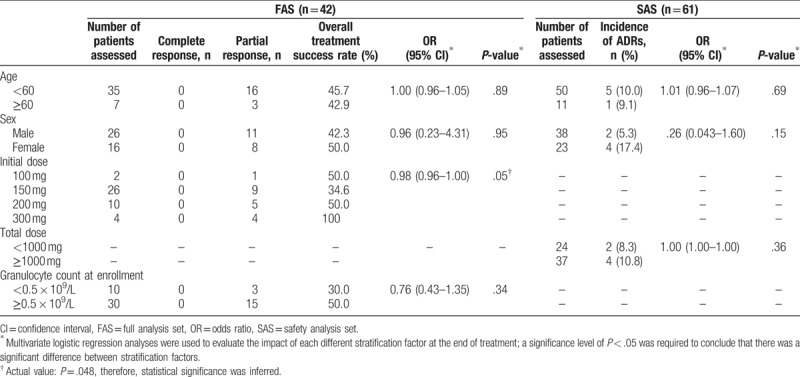
Results of post-hoc subgroup analyses to assess the impact of different stratification factors on the overall treatment success rate (FAS) and the incidence of ADRs (SAS).

None of the stratification factors assessed had a statistically significant impact on the incidence of ADRs in the SAS (Table 7).

## 4 Discussion

This study evaluated the efficacy and safety of micafungin in Chinese patients with probable or proven IA. For the primary endpoint, micafungin treatment resulted in overall treatment success in around 50% of patients; treatment success rates were higher in the PPS than in the FAS. Similar differences between the FAS and PPS were observed for secondary efficacy endpoints. These differences may be attributed to the exclusion from the PPS of patients who experienced TEAEs or SAEs (perhaps as a result of being unwell, less responsive to treatment, or having more severe illness) or were subject to study protocol violations.

Overall treatment success rates were lower than those derived from a post-marketing study assessing the effectiveness of micafungin against IA (70.8%).^[27]^ And also lower than those observed in clinical trials that assessed overall treatment success rates for micafungin against all IFDs (including IA) (range, 64.4–92.6%).^[15–17]^ However, when compared with studies conducted in a comparable patient population (i.e., patients with proven or probable IA), overall treatment success rates were similar to or higher than those previously reported for micafungin (range, 25.0–50.0%).^[19–21]^

Overall treatment success rates for micafungin were also within the range of those observed for caspofungin and voriconazole,^[21,28–34]^ and higher than those observed for amphotericin B,^[30,31,35,36]^ in several other studies of patients with proven or probable IA. However, such between-study comparisons should be made with caution, and some differences between these other studies and the current study should be noted. Firstly, the treatment duration in the other studies varied widely, from 7 days to 6 months. Secondly, patient demographics and characteristics were also varied between the other studies and differed from those reported in the current study; for example, none of these other studies were conducted in patients from China. Finally, the definitions reported for proven or probable IA, or measures of overall treatment success, although similar, were not identical to those used in the current study in several cases.^[21,28,35,36]^

TEAEs were observed in a high proportion (60.7%) of patients who received micafungin; this is consistent with the proportion observed in other clinical trials of micafungin for the treatment of IFDs,^[15–17]^ similar to those observed in patients receiving other treatments such as voriconazole or amphotericin B,^[31]^ but notably higher than in patients treated with caspofungin (including 1 comparative study).^[21,29,32–34]^ In other comparative studies, a similar safety profile has been reported for micafungin versus azole treatments (fluconazole and itraconazole).^[15–17]^ In the current study, decreased platelet count and fatigue were both reported in 8.2% of patients, a higher proportion than reported in previous trials, including those conducted in patients with proven or probable IA.^[15–17,19,21]^ Also, some vital signs and clinical laboratory results (e.g., respiratory rate, AST, and urea nitrogen levels) were reported as normal prior to treatment, but abnormal levels were recorded after treatment. While there is no immediately apparent reason that these TEAEs and abnormal vital signs were reported in the current study, they could perhaps be attributed to the medical profile observed in a significant proportion of patients at, or just prior to baseline (e.g., neutropenia, malignant blood disease, chronic obstructive pulmonary disease, and abnormal chest CT scan).

Overall, the findings from the current study add to the body of data that demonstrates that micafungin is effective in patients with IFDs, of which few studies were performed in this specific patient population with proven or probable IA. Current international guidelines for the diagnosis and management of IA recommend that echinocandins such as micafungin are used in settings in which azoles or polyene antifungals are contraindicated^[37]^ or in patients who are intolerant of azoles or have progressive disease.^[38]^ However, some of these recommendations are weak and based on moderate-quality evidence.^[37]^ Current evidence suggests that micafungin has similar efficacy and tolerability compared with caspofungin in this specific patient population.^[21]^ Further to this, a recently published review discussed the need for future comparative trials to evaluate micafungin treatment against standard antifungal therapy in patients with IA. Such trials, which ideally should be conducted in patients with proven or probable IA, will help to establish the exact role for micafungin within the range of currently available broad-spectrum antifungals.^[18]^

The current study was discontinued early because of low patient recruitment, due to difficulties screening patients (e.g., low numbers of patients positive for serum galactomannan antigen test) and collecting informed consent. The small sample size meant that although results in the subgroup analyses demonstrated statistical significance, the study was not sufficiently powered to show a significant difference between patients based on their initial doses of micafungin, or other stratification factors. Nonetheless, results provide some evidence that higher initial doses of micafungin (e.g., 300 mg compared with 150 mg OD) may be associated with higher overall treatment success rates (34.6% compared with 100% in the current study, respectively). Although evidence from pharmacokinetic (PK) analyses may not translate into clinical outcomes and should, therefore, be interpreted with caution, these data are supported by results from PK/pharmacodynamic (PD) studies conducted in adults in Japan with suspected *Aspergillus* or *Candida* infection. The results of these studies suggested that patients who received 200 to 250 mg/d (either as a dose of 250 mg OD, or as a twice-daily dose of 100 mg), had a 95% probability of maintaining a micafungin plasma concentration of 0.05 mg/L, thought to be effective against *Aspergillus* spp.^[39]^ However, a prospective study of intrapulmonary and plasma PK/PD in adult lung transplant recipients conducted in California, demonstrated that a lower micafungin dose of 150 mg OD was sufficient to maintain a minimally inhibitory concentration required to inhibit the growth of 90% of *A fumigatus* previously tested isolates (MIC_90_).^[40]^ Further studies should be conducted to establish the optimal initial/daily dose of micafungin, as well as the impact of other factors (e.g., sex, age, initial dose, daily dose, and granulocyte count) on the efficacy and safety of micafungin treatment, in patients with proven or probable IA.

The main strength of the study was the use of a clearly defined patient population (patients with proven or probable IA). Also, patients were treated according to their physician's usual clinical practice, even though the study was conducted within a controlled clinical trial setting. The main limitation of the study was the small sample size, which restricts the conclusions that can be drawn based on the study results. Few trials have provided head-to-head comparisons of efficacy and safety between micafungin and other treatments in patients with proven or probable IA^[20,21]^; one of these studies was also discontinued early due to low patient numbers and no clear conclusions could be drawn.^[20]^ Other limitations included the open-label study design and the lack of comparator treatments.

In summary, results from the current study suggest that micafungin is efficacious and well tolerated for the treatment of patients with proven or probable IA in China; similar overall treatment success and AE rates were observed compared with previous investigations of micafungin in patients with proven or probable IA. However, these findings are to be interpreted with care due to the small number of patients included in this study. Further comparative trials to confirm the efficacy and safety of micafungin in patients with proven or probable IA would be beneficial.

## Acknowledgments

The authors thank the patients and their families for participating in the study and to the following investigators: Liping Zhu, Jianmin Wang, Jun Ma, Ming Hou, Aining Sun, Jianda Hu, Jie Jin, Kang Yu, Xiaowu Tan, Peigen Gui, Shenghua Sun, Yuming Li, Jiulong Kuang, Linhua Yang, Li Liu, Xiaoyun Hu, and Jishi Wang.

Medical writing support, which was funded by Astellas Pharma Inc., was provided by David Griffiths, PhD of Bioscript Medical.

Leiyan Lv of Fan En Pharmaceutical Development Co. provided support from a statistical perspective, funded by Astellas Pharma Inc.
